# Quantifying passive myocardial stiffness and wall stress in heart failure patients using personalized ventricular mechanics

**DOI:** 10.1186/1532-429X-18-S1-O17

**Published:** 2016-01-27

**Authors:** Zhinuo Jenny Wang, Vicky Y Wang, Chris P Bradley, Martyn P Nash, Alistair Young, Jie J Cao

**Affiliations:** 1 grid.9654.e0000000403723343Auckland Bioengineering Institute, University of Auckland, Auckland, New Zealand; 2 grid.9654.e0000000403723343Department of Engineering Science, University of Auckland, Auckland, New Zealand; 3 grid.416387.fThe Heart Center, St Francis Hospital, Roslyn, NY USA; 4 grid.9654.e0000000403723343Department of Anatomy with Radiology, University of Auckland, Auckland, New Zealand

## Background

Heart failure (HF) patients present with a spectrum of phenotypes, including preserved ejection fraction (HFpEF) and reduced EF (HFrEF). The underlying mechanisms of HFpEF and HFrEF may be related to mechanical factors such as stiffness and stress. This study aimed to quantify passive mechanical properties in HFrEF and HFpEF patients using personalised left ventricular (LV) biomechanical analysis.

## Methods

Data from 9 HFrEF patients, 4 HFpEF patients, and 4 non-HF patients with normal LV function (control) were analyzed. LV finite element mechanical models were personalized using LV surface data segmented from cine cardiac magnetic resonance (CMR) images. All subjects underwent same day left and right cardiac catheterization. Beat-averaged intra-ventricular pressures were extracted from LV catheter traces and temporally aligned with the CMR images. A mechanical simulation of LV diastolic filling was optimized to each patient-specific geometry and pressure loading, incorporating non-linear myocardial anisotropic tissue behavior and myocyte fiber orientation derived from our published work. Global myocardial passive stiffness was estimated by optimization of the predicted LV surface displacements between diastasis and end-diastole to the observed CMR motion. Mid-ventricular end-diastolic fiber stress was also derived from the model.

## Results

Volume and pressure changes between diastasis and end-diastole, and LV mass measurements are shown in Table 1 (mass and volume data have been normalised by body surface area). Myocardial stiffness was significantly higher for the HFrEF group (mean ± SEM 7.6 ± 1.6 kPa) compared to both the control group (1.3 ± 0.3 kPa, p < 0.01), and the HFpEF group (2.1 ± 0.4 kPa, p < 0.01), but no significant difference was found between control and HFpEF groups due to small sample sizes. Mid-ventricular end-diastolic fiber stress was also significantly larger in the HFrEF group (3.3 ± 0.4 kPa) compared to control (1.0 ± 0.2 kPa, p < 0.001) and HFpEF patients (1.3 ± 0.2 kPa, p < 0.05).

**Table 1:** Clinical characteristics of the HFrEF, HFpEF, and Control groups.

Group	DS pressure (mmHg)	ED pressure (mmHg)	DS volume index (mL/m^2^)	ED volume index (mL/m^2^)	Ejection fraction (%)	LV mass index (g/m^2^)
HFrEF	15 ± 3*	26 ± 3*	127 ± 11*‡	147 ± 10*‡	24 ± 3*‡	123 ± 7*
HFpEF	11 ± 1†	18 ± 1†	47 ± 7	59 ± 6	67 ± 2	95 ± 14
Control	6 ± 1	11 ± 1	48 ± 2	65 ± 6	59 ± 3	76 ± 5

*p < 0.05 HFrEF vs. Control; †p < 0.05 HFpEF vs. Control; ‡p < 0.05 HFpEF vs. HFrEF. Abbreviations: diastasis (DS), end-diastole (ED), left ventricle (LV). Volumes and masses indexed to body surface area.

## Conclusions

Our findings suggest that personalized LV mechanical modeling may provide important diagnostic and therapeutic targets for HF management.

